# The Impact of Altered HCN1 Expression on Brain Function and Its Relationship with Epileptogenesis

**DOI:** 10.2174/1570159X21666230214110333

**Published:** 2023-08-15

**Authors:** Ke Zhao, Yinchao Li, Xiaofeng Yang, Liemin Zhou

**Affiliations:** 1 Department of Neurology, The Seventh Affliated Hospital of Sun Yet-sen University, No. 628, Zhenyuan Road, Xinhu Street, Guangming District, Shenzhen, China;; 2 Guangzhou Laboratory, Guangzhou, No. 9 XingDaoHuanBei Road, Guangzhou International Bio Island, Guangzhou 510005, Guangdong Province, China

**Keywords:** Hyperpolarization-activated cyclic nucleotide-gated cation channel 1, epilepsy, epileptogenesis, *I_h_* current, seizure threshold, pyramidal neurons

## Abstract

Hyperpolarization-activated cyclic nucleotide-gated cation channel 1 (HCN1) is predominantly expressed in neurons from the neocortex and hippocampus, two important regions related to epilepsy. Both animal models for epilepsy and epileptic patients show decreased HCN1 expression and HCN1-mediated *I_h_* current. It has been shown in neuroelectrophysiological experiments that a decreased *I_h_* current can increase neuronal excitability. However, some studies have shown that blocking the *I_h_* current *in vivo* can exert antiepileptic effects. This paradox raises an important question regarding the causal relationship between HCN1 alteration and epileptogenesis, which to date has not been elucidated. In this review, we summarize the literature related to HCN1 and epilepsy, aiming to find a possible explanation for this paradox, and explore the correlation between HCN1 and the mechanism of epileptogenesis. We analyze the alterations in the expression and distribution of HCN1 and the corresponding impact on brain function in epilepsy. In addition, we also discuss the effect of blocking *I_h_* on epilepsy symptoms. Addressing these issues will help to inspire new strategies to explore the relationship between HCN1 and epileptogenesis, and ultimately promote the development of new targets for epilepsy therapy.

## 1 INTRODUCTION

**Fig. (1):**
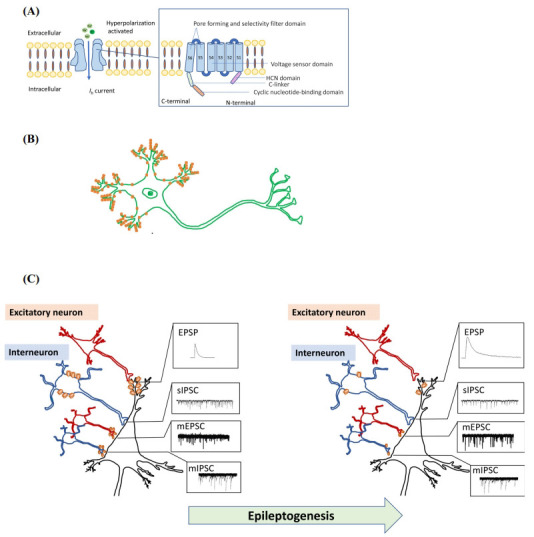
(**A**) Schematic illustration of an HCN channel and the structure of a subunit. HCN channels are tetrameric protein channels with permeability to Na^+^ and K^+^, and are activated by hyperpolarization of the membrane potential. Each isoform has a six-transmembrane domain topology. (**B**) Schematic illustration of the gradient distribution pattern of HCN1 channel in the pyramidal neuron dendrites. (**C**) Schematic illustration of the decreased HCN1 channels and the corresponding impact on neuron network excitability during epileptogenesis. When HCN1 channels and Ih decrease in the dendrites of pyramidal neurons, the excitatory input would produce EPSP with larger amplitude and longer duration in the dendrites. When HCN1 channels and Ih decrease on the soma of certain interneurons, the spontaneous firing of these interneurons would decrease, leading to reduced sIPSC frequency on the target neuron. When HCN1 channels and Ih decrease in the axons of certain excitatory neurons, the release of glutamate would increase, leading to increased mEPSC frequency on the target neuron. When HCN1 channels and Ih decrease in the axons of certain interneurons, the release of GABA would decrease, leading to reduced mIPSC frequency on the target neuron.

Hyperpolarization-activated cyclic nucleotide-gated cation channels (HCNs) are protein channels with permeability to Na+ and K^+^, activated by hyperpolarization of the membrane potential [1-3]. The current associated with HCNs is known as *I_h_* current [4] (Fig. **1A**). The HCN family has four members, HCN1, HCN2, HCN3, and HCN4 [2, 5]. HCN1 is the predominant isoform expressed in the neocortex and hippocampus, which are two important regions related to epilepsy [6-9]. Many studies have reported changes in HCN1 expression and distribution in the brain of animal models of epilepsy and in epileptic patients [10-17]. Moreover, mutations in the Hcn1 gene can cause epilepsy [18-22]. However, characterizing the impact that changes in HCN1 expression have on brain function is quite complicated, and many contradictory findings have been reported. For example, HCN1 expression and *I_h_* current are found to decrease in the dendrites of neocortical and hippocampal pyramidal cells, and decreased Ih current increases neuron network excitability [13, 14, 17, 23], whereas, surprisingly, blocking *I_h_* current increases the seizure threshold and improves seizure onsets [24, 25]. This paradox highlights how much is still unclear about the mechanism underlying HCN1 alterations and its association with epileptogenesis. In this review, we summarize the current research on the correlation between HCN1 and epileptogenesis and seizures, focusing on the following issues: What is the physiological function of HCN1? How do the functional changes affect neuronal excitability? How do the expression and distribution change in the course of epilepsy? What are the potential advantages of HCN1 as a therapeutic target for epilepsy? By answering these questions, we demonstrate the causal relationship between changes in HCN1 expression and distribution and epileptogenesis, and suggest new targets for the research and development of antiepileptic drugs and devices.

## 2 EXPRESSION, DISTRIBUTION AND PHYSIOLOGICAL FUNCTIONS OF HCN1

The expression and distribution of HCN1 are closely related to its physiological function. According to immunohistochemistry (IHC) results from the brains of adult male Sprague-Dawley rats, HCN1 is predominantly distributed in the cortical region, with the staining being intense in the neocortex, hippocampus, superior colliculus, and cerebellum [7]. At the cellular level, single-cell RNA sequencing analysis of HCN1 expression in the mouse brain showed that among cortical cell types, PV+ interneurons and pyramidal neurons have a high level of expression [26]. In the hippocampus, HCN1 is mostly located in CA1 pyramidal cells and oriens-lacunosum-moleculare interneurons (inhibitory interneuron located in stratum oriens, stratum lacunosum, and stratum moleculare in CA1 [27]). In terms of the subcellular distribution, the predominant location of HCN1 varies among different types of cells. It can be located on dendrites, soma or axon, and participate in various physiological activities [28].

Since the function of HCN channels is carried out *via* the *I_h_* current, many studies have focused on this current to explore the physiological function of HCN channels. HCN subunits can form both homomeric and heteromeric channels in the brain, and HCN1, in particular, can form a heteromeric channel with HCN2 in the neocortex and the hippocampus. However, when different HCN subunits co-exist in a single cell, the composition of HCN channels is mainly dominated by one of these subunits [7, 26]. We measured the expression of HCN2 in the rat frontal neocortex and hippocampus *via* IHC, and observed it to be considerably lower than that of HCN1. Therefore, since HCN1 appears to be the predominant subunit in these brain regions, the *I_h_* current could be considered to largely represent the function of HCN1, and many physiological functions of HCN1 will be discussed in the following paragraphs in terms of *I_h_* current.

The typical subcellular location of HCN1 is in the dendrites of pyramidal neurons, with a gradient-like distribution pattern along the dendrite axis in which the channel is enriched at the distal end [7, 29, 30] (Fig. **1B**). Since a small fraction of HCN channels remains open at resting membrane potential (RMP), *I_h_* in dendrites can reduce membrane resistance [31]. When a series of synaptic inputs produce excitatory postsynaptic potential (EPSP) in the dendrite membrane, the *I_h_* current can influence the amplitude and shape of synaptic input, reducing EPSP summation and membrane excitability [30, 32]. This process is always described as the ‘dampening of the excitatory input’ [30]. The *I_h_* current is also involved in the processing of inhibitory postsynaptic potential (IPSP). The *I_h_* current normalizes the impact of dendrite IPSP on the soma, regardless of the distance between the site of the IPSP and the soma [33]. But it needs to be noted that these phenomenona are actually the net effects of the activity of different channels, with HCN1 acting in combination with Ca^2+^ and K^+^ channels [34, 35].

In cortical and hippocampal interneurons, HCN channels localize preferentially in the somatic region of the cell, where they co-regulate cellular properties like the resting membrane potential and the spontaneous firing frequency [36]. HCN1 and HCN2 are also located in the stratum oriens of CA1, which is where the cell body and proximal dendrites of interneurons are located [7, 37]. Here, the *I_h_* current contributes to the generation of spontaneous action potential firing in these interneurons [38]. When ZD7288 (a HCN channels blocker) was applied to these spontaneously firing cells in the absence of tonic current injection, a reduction in the action potential frequency was observed. A similar finding is that inhibition of the *I_h_* current in the CA1 stratum orient layer could lead to reduced spontaneous inhibitory postsynaptic currents (sIPSCs) in CA1 pyramidal cells (the targets of these interneurons) [39].

HCN1 channels are also present on certain axons and synaptic terminals, where they are involved in neurotransmitter release [40-42]. HCN1 channel exists pre-synaptically in a subset of synaptic terminals targeting entorhinal cortical layer III pyramidal neurons within the mature entorhinal cortex (EC) of the mouse, and there it decreases the release of glutamate by interacting with T-type Ca^2+^ channels [41, 42]. HCN1 may also be involved in the regulation of GABA release [40]. HCN channels are expressed in the basket cells (BCs) of the dentate gyrus at both somatodendritic and axonal compartments. Blocking of the *I_h_* current in BCs from the dentate gyrus can reduce the frequency of miniature inhibitory postsynaptic currents (mIPSCs) in granule cells, implying that HCN1 can facilitate neurotransmitter release from inhibitory terminals [40]. F.C. Roth and H. Hu also found that functional HCN channels in PV+-BCs are exclusively expressed in axons, being completely absent from the soma and dendrites, and they enhance AP initiation and speed up the propagation of AP trains in these axons [43].

## 3 CHANGES IN HCN1 EXPRESSION AND DISTRIBUTION IN EPILEPSY AND THEIR EFFECTS ON NEURONAL EXCITABILITY

A large number of experiments have demonstrated that the total protein expression of HCN1 is decreased in animal models of epilepsy and epileptic patients. Shah *et al.* found HCN1 and HCN2 to be down-regulated in EC tissue 24 hours after kainic acid (KA)-induced status epilepticus (SE), which corresponds to a reduction in dendritic *I_h_* current in EC-III-pyramidal cells [13]. In their study, depolarizing current injections into EC pyramidal cells induced a larger number of action potentials after SE, indicating increased cell excitability and resulting in increased excitatory drive to the hippocampus that may contribute to epileptogenesis. Similar decreases in the levels of HCN1 protein were also reported in the CA1 region of the hippocampus in a pilocarpine-induced rat model of SE both in the acute (less than 1 week after pilocarpine administration) and chronic periods (2 weeks after SE induction, when rats were described as gradually exhibiting spontaneous seizures) [14]. However, in this research, *I_h_* current was found to decrease in dendrites in this study, whereas soma *I_h_* current remained unaltered. When the authors injected a depolarizing current into the dendrites of pyramidal neurons, there was a significantly higher frequency of action potential (AP) firing compared to control animals, indicating increased excitability of these dendrites. Total HCN1 protein was also found to decrease in the hippocampus of human medial temporal lobe epilepsy (MTLE) patients [16]. Hyperthermia-induced SE was also associated with decreased HCN1 expression in a febrile seizure (FS) rat model [11]. Only in this model, the *I_h_* current was enhanced, possibly because HCN2 mRNA was found to be increased and may have formed more heteromeric channels with HCN1 both on the soma and dendrites of pyramidal cells [10, 11, 44]. After FS, GABA-mediated inhibition is increased, while at the same time, the enhanced *I_h_* current antagonizes the repetitive inhibitory inputs and can convert them to post-inhibitory rebound firing in CA1 pyramidal cells, contributing to a decreased seizure threshold after FS [10]. Dyhrfjeld-Johnsen *et al.* tried to explain enhanced *I_h_* and neuron excitability from an *in vitro* electrophysiological perspective [45]. However, one thing should be noticed: although in this study, HCN1 expression decreased in all FS rats, not all of them developed epileptic symptoms in adulthood. Therefore, more studies are needed to confirm the link between increased *I_h_* current and epileptogenesis [46]. Sometimes the total protein levels of HCN1 may not represent well the actual amount of functional channels located on the membrane. For example, Shin *et al.* found that in the KA-induced rat model, the total protein levels of HCN1 remained unchanged after SE, but the levels of membrane protein actually increased 24 hrs after SE, and then decreased during the chronic period (28 d after SE) [17]. The authors pointed out that it is the level of membrane protein that actually underlies the change in physiological function. The changes related to HCN1 in epilepsy also involve a change in the distribution pattern, also known as channel re-localization. Just as the total protein level of a given tissue cannot represent the HCN1 function on that tissue, the HCN1 expression levels in the membrane cannot by themselves reflect its distribution pattern, with the function of HCN1 channels depending on both [47]. In the study by Shin *et al.*, in which HCN1 protein levels in the membrane were found to decrease, the enrichment of HCN1 in the distal end of the dendrites was also not observed. In addition, HCN1 expression increased in the stratum pyramidale (SP) layer, where the soma of pyramid neuron is located, implying that the original distribution of HCN1 changed in the course of epilepsy [17]. HCN1 is also expressed in astrocytes and microglial cells, and participates in the pathophysiological changes that underlie many diseases [48, 49], but its physiological functions in these cells and the potential changes in its levels of expression related to epilepsy are yet to be studied.

## 4 THE REGULATORY MECHANISMS OF HCN1 IN EPILEPSY DEVELOPMENT

Many studies imply that excitatory stimulation can increase HCN1 expression. For example, Fan *et al.* found that in hippocampal slices, potentiated synaptic inputs can upregulate the expression of HCN1 and enhance *I_h_* current in CA1 pyramidal neurons, thus reducing their excitability [50]. They postulated that this is a kind of negative feedback in response to long-term potentiation (LTP) whose purpose is to maintain network stability [50]. Noam *et al.* reported similar findings in culture experiments with hippocampal neurons from rats. The application of glutamate could activate NMDA or AMPA-type ionotropic glutamate receptors, thus inhibiting HCN1 trafficking and resulting in increased surface expression of HCN1 on neuronal cells [51]. Brager and Johnston found that long-term depression (LTD) at Schaffer collaterals could downregulate *I_h_* on the dendrites of the CA1 pyramidal neuron, which proved the relationship between LTP and HCN1 from another angle [52]. However, Richichi *et al.* reported a contradictory finding: activated Ca^2+^-permeable AMPA receptors and the subsequent activation of CaMKII trigger the reduction, rather than the upregulation, of HCN1 expression [53]. In this study, seizure-like events were generated using the glutamate receptor agonist KA in a culture system consisting of P8 organotypic hippocampus slices from rats. The selective Ca^2+^-permeable AMPA-receptor blocker NASPM abolished the downregulation of HCN1 expression induced by seizure activity. But these authors did not measure the *I_h_* current, although this can be assumed to represent the actual amount of functionally available HCN1 channels on the membrane [17]. The establishment and maintenance of HCN1 enrichment in the distal end of the dendrites are also regulated in the course of epilepsy. During growth and development, this HCN1 enrichment is related to glutamate receptor activity and Ca^2+^/calmodulin-dependent protein kinase II (CaMKII) [54]. The chaperone protein Trip8b is also involved in HCN1 trafficking and expression in the distal membrane of dendrites [55-57]. In the KA-induced rat model of epilepsy, the levels of TRIP8b Ser237 phosphorylation and CaMKIIα activity are reduced after SE induction, which leads to reduced TRIP8b-HCN1 binding, resulting in a disrupted HCN1 gradient from soma to dendrite, and this may be involved in epileptogenesis [58]. Still, whether increasing the phosphorylation at Ser237 can restore the physical distribution pattern of HCN1 and relieve seizure is not yet known. A large number of studies have demonstrated that the omics-level changes related to the expression and distribution of HCN1 also affect epileptogenesis, progression, and prognosis of epilepsy. McClelland *et al.* reported that SE can augment the binding of the transcriptional repressor NRSF (neuron-restrictive silencer factor REST) to the Hcn1 gene, resulting in suppression of HCN1 expression and *I_h_* current in dendrites from hippocampal CA1 pyramidal cells [59]. Zha *et al.* reported that seizure-like activity (SA) can increase the abundance of glycosylated HCN1, leading to increased HCN1/HCN2 heteromerization in the hippocampus *in vivo* as well as in hippocampal organotypic slice cultures [60]. The increased formation of heteromeric channels is associated with altered properties of the resulting *I_h_* current, significantly enhancing network excitability [10, 60]. Phosphorylation is also one of the mechanisms regulating *I_h_* current properties and HCN1 surface expression in normal physiological conditions [61]. Concepcion *et al.* identified numerous phosphosites of HCN1 in hippocampal tissues from a TLE rat model and in resected human brain tissue containing epileptogenic zones (EZs); some of the changes replicated the altered properties of the *I_h_* current, indicating that HCN1 channel phosphorylation may be of importance in the pathogenesis of epilepsy [62]. Many accessory proteins are also involved in regulating HCN channel properties, and are therefore also relevant for the mechanisms of epileptogenesis [18, 63].

## 5 HCN1 AS A THERAPEUTIC TARGET IN EPILEPSY

If the hypothesis from Fan *et al.* [50] about increased membrane expression of HCN1 in pyramidal neurons being a kind of negative feedback in response to increased excitatory input to maintain network stability turns out to be correct, it is possible that the decreased HCN1 and *I_h_* current of CA1 pyramidal cells in epilepsy refers to the failed compensation to increased excitatory inputs, and increasing HCN1 or *I_h_* current may have value as therapeutic targets for epilepsy. So far, only a single study has been consistent with this possibility. McClelland *et al.* found that in the KA-induced rat model, the expression of Hcn1 and many other genes containing NRSF (neuron-restrictive silencer factor) binding sequence was repressed. The use of oligodeoxynucleotides (ODNs) to block the binding during the two weeks after SE can increase the expression of HCN1 and *I_h_* current function, and can significantly reduce the number of seizures in the chronic period [59]. Although HCN1 is just one of several hundred neuronal genes that contain the binding sequence and respond to the ODNs, it is still possible that reversing the downregulation of HCN1 and the *I_h_* current may be beneficial to epilepsy treatment.

At the same time, there are some controversial findings about the implications of targeting HCN1 as a therapeutic strategy (Table **1**). Luszczki and Cavalcante found that blocking the *I_h_* current in the whole brain will decrease the susceptibility to seizures and also their severity. Ivabradine is a drug used to treat arrhythmia and heart failure, and when administered, it blocks HCN channels in the heart. Intraperitoneal injection of ivabradine was found to elevate the threshold for maximal electroshock-induced tonic seizures in mice [24]. Intraperitoneal injection of ivabradine could also attenuate pentylenetetrazole (PTZ) and pilocarpine (PILO) induced seizures in mice [25]. Since ivabradine does not pass through the blood-brain barrier [72], it was orally administered in another study after injection of the P-glycoprotein inhibitor elacridar (which can increase its penetration by inhibiting the efflux transporter). The authors reported a marked and dose-dependent decrease in absence seizures (ASs) displayed by Genetic Absence Epilepsy Rats From Strasbourg (GAERSs) [68].

ZD7288 is a frequently used type of HCN blocker that also seems to have anti-epileptic effects. ZD7288 can decrease epileptiform hyperexcitability in rat neocortex slices induced by 4-AP [64]. Local intracranial injection of ZD7288 can significantly increase the threshold of electrically-induced paroxysmal discharges in rabbits [69]. Oral administration of ZD7288 was also reported to have anti-epileptic effects on Mongolian gerbils with inherited epilepsy [70].

However, neither ivabradine nor ZD7288 are specific HCN1 blockers. The anticonvulsant effect of ivabradine may also be related to its high affinity for the GABAA receptor [25]. Additionally, both ivabradine and ZD7288 block HCN4, a subunit predominantly located in the thalamus [7, 73]. In the thalamus, HCN4 channels are involved in the generation of pacemaker activity, which is critical for maintaining hypersynchronized neuronal network activity during seizures [74, 75]. The HCN4-specific blocker EC18 has already been reported to be able to decrease seizure susceptibility and neuronal network excitability in adult mice [76]. Thus, the clinical effectiveness of ZD7288 and ivabradine is not sufficient to prove the benefits of blocking HCN1 channels for epilepsy treatment.

Only a few chemical compounds have been reported to be able to selectively block HCN1 channels, including (R)-6 (also referred to as MEL57A in another search from the same research team) [77, 78], and a compound termed 12m [79]. MEL57A was reported to have no effect on seizure susceptibility in both the subcutaneous PTZ seizure assay and the thermogenic seizure assay [71]. However, in this research, MEL57A was administrated to mice intraperitoneally in that study, and the authors did not assess the ability of the drug to pass through the blood-brain barrier.

In conclusion, none of these findings can prove with certainty that selective blocking of HCN1 channels in the brain has a beneficial effect on epilepsy treatment. Hence, specific HCN1 blockers should be tested before or after spontaneous seizure onset to accurately evaluate the involvement of HCN1 channels in epileptogenesis, and their suitability as a target for epilepsy therapy.

## CONCLUSION AND FUTURE PROSPECT

In summary, the physiological functions of HCN1 channels can be summarized as decreasing the effect of excitatory inputs while increasing the release of the inhibitory signal (Fig. **1C**). When the expression of HCN1 is downregulated in pyramidal neurons or interneurons, the balance of excitation and inhibition is shifted toward excitation. So far, no study has conclusively proven that decreasing HCN1 protein levels or blocking HCN1 channels result in an antiepileptic effect. From this perspective, it is reasonable to consider that the decreased expression of HCN1 in the hippocampus and neocortex observed in epileptic animals and patients may be related to the cause of epileptogenesis. To further clarify this causal relationship, it needs to be demonstrated that decreased HCN1 expression in the hippocampus and neocortex or specific blockage of HCN1 channel can increase the susceptibility to epilepsy. A particular issue that needs to be considered in future studies that aim to evaluate the effect of increasing HCN1 expression on specific brain areas to treat epilepsy is to finely regulate the amount of HCH1 protein. Since upregulated *I_h_* can increase neuron excitability, the aim of therapy should be to bring HCN1 levels back to those found under physiological conditions. Besides the total protein expression level, the subcellular distribution of HCN1 may be of equal importance. The gradient distribution of HCN1 along the soma-axon axis in the pyramidal neuron is closely related to the physiological function of HCN1 channels. Hence, it needs to be investigated whether a disrupted distribution pattern can aggravate epilepsy and whether re-creating this pattern can improve symptoms or prevent epilepsy. The specific changes in HCN1 expression among different cell types and the corresponding impacts on epileptogenesis also need to be studied. Cell-specific virus infection can be a useful tool to achieve this. A specific HCN1 blocker is also needed to explore the role of HCN1 channels in epilepsy therapy. Furthermore, the net effect of changed HCN1 and other co-expressed channels within the same group of cell should be considered when interpreting the impact of altered HCN1 expression in the brain. In conclusion, more details related to HCN1 alterations and the corresponding net impacts on epileptogenesis need to be researched so as to form a complete picture of how changes in HCN1 expression may lead to epilepsy that could ultimately help with the development of new targets for epilepsy therapy.

## Figures and Tables

**Table 1:** Studies involving various epilepsy models with HCN1 or *Ih* as therapeutic target.

**References**	**Experimental Epilepsy Models**	**Animals**	**Drug**	**Administration**	**Results**
***In vitro***					
Inaba *et al.* [64]	Neocortex slices induced by 4-AP	Male Wistar rats (300-350 g)	ZD7288	Bath	ZD7288 decreases epileptiform hyperexcitability
Albertson *et al.* [65]	Neocortex slices induced by bicuculline	20-26 days rats	ZD7288	Bath	ZD7288 increased the magnitude of evoked epileptiform events
Arias *et al.* [66]	Hippocampal slices induced by low Mg^2+^/high K^+^	SD rats (250g)	ZD7288	Bath	ZD7288 blocked spontaneous bursting activity
Gill *et al.* [67]	Hippocampal slices induced by low Ca^2+^/elevated K^+^, low Mg^2+^/elevated K^+^, or bicuculline.	Adult male Lister-Hooded rats	ZD7288	Bath	ZD7288 inhibited epileptiform bursting
***In vivo***					
Luszczki *et al.* [24]	Maximal electroshock-induced tonic seizures model	Male Swiss mice (22-26 g)	Ivabradine	Intraperitoneal injection	Ivabradine elevated the threshold of tonic seizures
Cavalcante *et al.* [25]	PTZ and PILO model	Male Swiss mice (25-30 g)	Ivabradine	Intraperitoneal injection	Ivabradine attenuated seizures
McClelland *et al.* [59]	KA model	Male Wistar-Han rats	ODNs	Intracerebroventricular injection	ODNs increased HCN1 expression and reduced the number of seizures in the chronic period
Iacone *et al.* [68]	GAERSs	Adult male GAERSs (250-300 g)	Ivabradine	Per os	Ivabradine decreased the number of absent seizures
Kitayama *et al.* [69]	Electrically-induced model	Adult male rabbits (2.4-3.2 kg)	ZD7288	Local intracranial injection	ZD7288 increased the threshold of paroxysmal discharges
Matsuda *et al.* [70]	Inherited epilepsy Mongolian gerbil model	Adult Mongolian gerbils	ZD7288	Per os	ZD7288 had anti-epileptic effects
Kharouf *et al.* [71]	PTZ model Hyperthermic seizure model	C57BL/6J post-weaning (P21-28) male mice	Ivabradine MEL57A	Intraperitoneal injection	Ivabradine reduced seizure susceptibility in the s.c.PTZ seizure assay and thermogenic assay MEL57A had no effect on seizure susceptibility

## LIST OF ABBREVIATIONS

ASs: Absence seizures
BCs: Basket Cells
CaMKII: Ca^2+^/Calmodulin-dependent Protein Kinase II
EC: Entorhinal Cortex
EPSP: Excitatory Postsynaptic Potential
FS: Febrile Seizure
GAERSs: Genetic Absence Epilepsy Rats From Strasbourg
HCN1: Hyperpolarization-activated Cyclic Nucleotide-gated Cation Channel 1
IHC: Immunohistochemistry
IPSP: Inhibitory Postsynaptic Potential
KA: Kainic Acid
LTD: Long-term Depression
LTP: Long-term Potentiation
mIPSCs: Miniature Inhibitory Postsynaptic Currents
MTLE: Medial Temporal Lobe Epilepsy
NRSF: Neuron-restrictive Silencer Factor
ODNs: Oligodeoxynucleotides
PILO: Pilocarpine
PTZ: Pentylenetetrazole (PTZ)
SE: Status Epilepticus
sIPSCs: Spontaneous Inhibitory Postsynaptic Currents
SP: Stratum Pyramidale Layer
